# Understanding quality improvement at scale in general practice: a qualitative evaluation of a COPD improvement programme

**DOI:** 10.3399/bjgp14X682801

**Published:** 2014-12-01

**Authors:** Martin Marshall, James Mountford, Kirsten Gamet, Gulsen Gungor, Conor Burke, Robyn Hudson, Steve Morris, Nishma Patel, Phil Koczan, Rob Meaker, Cyril Chantler, Christopher Michael Roberts

**Affiliations:** University College London, London.; UCLPartners, London.; Patients Know Best, Cambridge.; NHS Redbridge Clinical Commissioning Group, Ilford.; Barking and Dagenham, Redbridge and Havering Clinical Commissioning Groups, Romford.; Peter MacCallum Cancer Centre, Melbourne, Australia.; University College London, London.; University College London, London.; UCLPartners, London.; Barking and Dagenham, Redbridge and Havering Clinical Commissioning Groups, Romford.; UCLPartners, London.; UCLPartners and consultant respiratory physician, Barts Health NHS Trust, London.

**Keywords:** chronic obstructive pulmonary disease, general practice, large-scale improvement

## Abstract

**Background:**

A growing body of knowledge exists to guide efforts to improve the organisation and delivery of health care, most of which is based on work carried out in hospitals. It is uncertain how transferable this knowledge is to primary care.

**Aim:**

To understand the enablers and constraints to implementing a large-scale quality improvement programme in general practice, designed to improve care for people with chronic obstructive pulmonary disease.

**Design and setting:**

A qualitative study of 189 general practices in a socioeconomically and ethnically-mixed, urban area in east London, UK.

**Method:**

Twelve semi-structured interviews were conducted with people leading the programme and 17 in-depth interviews with those participating in it. Participants were local health system leaders, clinicians, and managers. A theoretical framework derived from evidence-based guidance for improvement programmes was used to interpret the findings. A complex improvement intervention took place with social and technical elements including training and mentorship, guidance, analytical tools, and data feedback.

**Results:**

Practice staff wanted to participate in and learn from well-designed collaborative improvement projects. Nevertheless, there were limitations in the capacities and capabilities of the workforce to undertake systematic improvement, significant problems with access to and the quality of data, and tensions between the narrative-based generalist orientation of many primary care clinicians and the quantitative single-disease orientation that has characterised much of the quality improvement movement to date.

**Conclusion:**

Improvement guidance derived largely from hospital-based studies is, for the most part, applicable to improvement efforts in primary care settings, although large-scale change in general practice presents some particular challenges. These need to be better understood and addressed if improvement initiatives are to be effective.

## INTRODUCTION

In recent years a growing body of research evidence has emerged describing the actions required to achieve substantive and sustained improvements in the organisation and delivery of clinical care. Studies have identified a number of high-level factors that appear to be associated with effective improvements, including the need for a shared vision and strategic capacity,^1^ leadership,^2^ a workforce with capacity and capability to deliver,^3^ the use of theoretically sound change models,^4^ and investment in infrastructure particularly relating to the use of data.^5^

The evidence has been brought together in the form of practical guidance for teams and organisations embarking on improvement initiatives.^6^ The authors of this guidance identified 10 challenges, categorised within three themes: design and planning, organisational context and leadership, and spreading and sustaining improvement (Box 1). The guidance appears straightforward but experience suggests that its implementation in practice is unlikely to be so. This is particularly the case in general practice, which, in comparison with the hospital sector, is relatively new to large-scale improvement efforts.^7^,^8^ Compared with the hospital sector, organisational entities in general practice are small, heterogeneous, and less well networked, the staff have fewer opportunities to learn about improvement methods, systems are often less well developed, and the infrastructure and governance arrangements to support improvement less mature.

Box 2. **Framework to guide effective improvement**

**Table:** 

**Theme**	**Challenge**	**Components**
**Design and planning**	Convincing people that there is a problem	Demonstrate the existence and scale of the challenge by using hard data and patient narratives Encourage peer-led debate and challenge Convince people that the problem is actionable
	Convincing people that the solution chosen is the right one	Present relevant research evidence and encourage discussion about its local applicability Make a commitment to evaluating the impact of the programme Use models and theories to demonstrate how your solution is likely to work Support local peer champions
	Getting data collection and monitoring systems right	Invest in data collection and analytical systems Expect and respond to criticisms of data quality Commit to auditing data quality Build capability among staff to utilise data
	Being realistic	Avoid hyperbole and evangelism Have high expectations but match ambitions to resources and capabilities
**Organisational context and leadership**	Creating a conducive environment for change	Align improvement goals with strategic objectives Present a motivating vision and rationale for change Create a secure environment for change Promote a commitment to learning and professional development
	Engaging staff	Clarify who owns the problems and solutions, and the need to work across traditional boundaries Use language that engages all stakeholders Create time and space for all people to think and work differently
	Promoting effective models of leadership	Clearly describe models of leadership appropriate to the task, emphasising the importance of enabling and facilitation skills Identify and support leaders
	Managing change by achieving a balance between positive incentives and sanctions	Emphasise, enable, and promote the intrinsic professional motivation of staff Use positive external incentives where required and align them to intrinsic motivators Be prepared to actively manage performance when required
**Spreading and sustaining improvement**	Embedding change and a commitment to continuous improvement	‘Future-proof’ improvement initiatives by identifying long-term leadership and allocating the necessary resources Embed success into standardised ways of working
	Considering the side effects of change	Be open to the unintended consequences of change Predict and where possible manage these effects

Adapted from the Health Foundation (2012).^6^

This study describes the results of the qualitative component of a multi-method evaluation of a large-scale initiative based in primary care that aimed to improve outcomes for people with chronic obstructive pulmonary disease (COPD). The study aimed to explore the perceptions of the programme among local health system leaders and front-line clinicians, and the facilitators and barriers to its implementation, and thereby to draw general lessons about improvement initiatives involving multiple general practices. The scale and nature of the project enabled the research team to explore the applicability of widely agreed ‘best practice’ improvement guidelines within the grounded reality of general practice.

**How this fits in**

Much of what is known about systematically improving the organisation and delivery of health care is derived from research and practice carried out in the hospital sector. The applicability of this learning to improvement efforts in general practice and primary care settings is unclear. The culture, structures, and ways of working of general practice present a new set of challenges to those responsible for health system improvement. Efforts to improve general practice will not be successful unless the enablers and constraints to systematic improvement in the sector are better understood.

## METHOD

A qualitative study was undertaken using semi-structured and in-depth interviews with local health system managers, GPs, and practice nurses.

### Setting and participants

The improvement programme was carried out between April 2011 and May 2012 in 189 general practices across four boroughs (administrative localities) in outer northeast London. The area is socioeconomically and ethnically mixed, with some highly deprived communities. COPD is known to be common, underdiagnosed, and often suboptimally managed.^9^ At the start of the study 2702 patients were registered as having COPD, though national data suggest that up to two-thirds of people with COPD are undiagnosed.^9^

The evaluation was carried out in two phases. First, 12 face-to-face semi-structured interviews were conducted with health system leaders (non-clinical and clinical executive directors of the primary care trusts that commission and manage health services) from the four boroughs in outer north-east London, and with the individuals responsible for delivering the different elements of the intervention to the practice teams. Second, 17 in-depth interviews were conducted with front-line primary care staff, including GPs, practice nurses, and practice managers. For each phase, a purposive and snowball sample of participants was selected, attempting to represent as wide a range of views as possible.

### Intervention

A complex set of interventions with both social and technical elements was co-designed by a team of primary and secondary care clinicians, managers, researchers, and service users. The different elements of the set were based on accepted best practice, drawing on the published research evidence and professional consensus.^10^,^11^ The intervention comprised five elements: four 0.5-day master-classes delivered by a small team of specialists; a 1.5-day course of spirometry training delivered by an accredited trainer and resulting in a certificate of competency; mentorship for practice nurses provided by a specialist nurse, in the form of a short discussion followed by an opportunity to work together in a clinic; infrastructure support for the practices in the form of a template to support data extraction and a patient self-management plan; and data feedback in the form of dashboards presenting the performance of the practices across a number of indicators derived from national guidelines produced by the National Institute for Health and Care Excellence (NICE) and COPD-related hospital admissions, and benchmarking practice performance against aggregate scores for all of the practices involved. All elements of the intervention were provided free of charge to the practices. The dashboards were created by a small analytics vendor, based on data extracted directly from the patient records and routine hospital statistics.

### Data collection

The semi-structured interviews with system leaders and those who delivered the interventions were conducted in person and lasted about 1 hour. A brief background information paper and an interview schedule designed to explore perceptions of the programme and the barriers and facilitators to its implementation were sent to the participants prior to the interviews. The in-depth interviews with front-line staff from across the practices probed more deeply into the motivations and attitudes of clinicians and managers to the programme. Interviews lasted between 30 minutes and 1 hour, and were carried out by an independent contract researcher supported by two authors. The interviews were conducted at a location convenient for the participants and the interviews were audiorecorded and fully transcribed.

### Data analysis and interpretation

A simple thematic analysis of early interview transcripts was carried out in an iterative fashion, so that emerging themes could be fed back into, and tested out in, subsequent interviews. In line with the aims of the study, the 10 ‘challenges’ from the published guidance for large-scale improvement initiatives (Box 1) were used deductively as a framework and as a programme-level theory to structure the analytical process.^12^ The emergent themes from the data were found to readily align to this framework, though some of the challenges were addressed in more detail than others. The analytical and interpretative process was conducted iteratively by three authors moving themes and sub-themes between the framework challenges until coherent response patterns emerged. These patterns were then discussed and modified at team meetings. Direct quotations were taken from the transcripts to illustrate key themes.

## RESULTS

The results are presented in the form of the 10 key challenges for large-scale improvement:

### Challenge 1: Convincing people that there is a problem

None of the interviewees were defensive about deficiencies in the current management of COPD, or doubted that outcomes for patients could be improved. They did not appear to need data to convince them that there was a problem, preferring to base their opinions on their own personal experience or on stories told by colleagues. However, it was clear that recognising that there was a problem would not necessarily lead to concrete action. Many of the interviewees did not see COPD as having a higher priority than many other areas of their clinical practice, as one responder described when approached to participate in the work:

‘I’d like to speak to you about COPD. I’d also like to speak to you about integrated case management for long-term conditions, chronic heart failure, telehealth. Oh, and by the way … The agenda for the CCG … is longer than my arm.’

(System leader)

### Challenge 2: Convincing people that the solution chosen is the right one

Most of the responders were positive about the solutions chosen by the project team to address the problem. They welcomed a structured and systematic approach to improving COPD care. They also expressed positive views about the multifaceted nature of the intervention, contrasting it with what they perceived to be a narrow and technocratic approach to change exemplified by the use of financial incentives. They found the educational components of the package to be most useful, and commented positively on the involvement of a local specialist who had an international reputation in the field. The data analytics element was perceived to be least useful.

### Challenge 3: Getting data collection and monitoring systems right

The data components of the project presented significant problems to the responders. Negative views were expressed about the inter-operability of the different medical record systems in place, the delay in implementing a common template for data extraction at the start of the project, the poor quality of data coding, the weak linkage with other relevant datasets, and the length of time it took to receive data from the hospital. Some responders found the data that was fed back to the practices early in the project to be too dense and complex:

*‘I must say, there are too many statistics. If our practice manager can’t understand* [the analytics model used], *how are we going to understand it? So, it’s just too much maths, too many statistics.’*

(GP)

Concerns were expressed about both the capacity and the capability of primary care teams to utilise data effectively, with a suggestion that many staff working in general practice lacked the basic skills required to analyse and interpret data:

*‘We only have one IT person in the whole practice and she is busy … just running the QOF* [Quality and Outcomes Framework] *things.’*

(Practice nurse)

### Challenge 4: Being realistic

The interviewees were complimentary about the extent to which the project was grounded in the realities of front-line general practice, particularly in relation to the time pressures on the people who needed to be engaged with the work:

‘They’re really busy people so you need to make everything really painless for them.’

(System leader)

A wide range of challenges faced by the practices were discussed. These included: the difficulty experienced by staff in making time to be proactive; the increasing part-time, temporary, and shift-working patterns of the workforce; the need for ‘just in time’ learning; the capabilities of the staff in relation to new areas of practice such as the use of self-management plans; the demographics of the population; scepticism about the external imposition of change; and the unique challenges of small practices. Each of these was perceived by the responders to have been understood and dealt with sensitively by the project team.

### Challenge 5: Creating a conducive environment for change

The system leaders expressed concerns that many of the participating practices did not have a culture that supported systematic quality improvement work, or one that valued collaboration with other practices. Several of the responders described how the geographical spread and the structural and procedural heterogeneity of general practices made it difficult to even communicate with the practice staff, never mind convey a common vision or implement a shared plan.

### Challenge 6: Engaging staff

The interviewees felt that the practical problems summarised in Challenge 4 should be, and mostly were, addressed by the project team in a number of ways. First, primary care teams were considered to be more responsive to the social elements of improvement activities than to technical ones — a desire to be informed by narrative data more than numeric data, a preference for flexibility over directives, and for peer support and challenge over managerialism:

*‘With GP consortia there are practices that do participate more in things because they have to answer to their peers, as opposed to answering to the PCT* [primary care trust, the administrative body in primary care].’

(System leader)

Second, the varying needs of different members of the primary care team were recognised. For example, some practice nurses with special interests were perceived to be motivated by more exacting standards (such as NICE guidelines) than their GPs (who were oriented to QOF) and to be more committed to attending educational courses, obtaining formal qualifications, and utilising mentoring opportunities. Third, the importance of conveying a bigger vision was described by the responders, a sense of excitement and describing new, proactive ways of working to front-line staff, many of whom expressed negative views about their work:

‘In general practice … we are trying to fight fires all the time …’

(GP)

Fourth, the importance of starting where people are, rather than where it is perceived that they should be, was widely described. For example, it was considered important to recognise that many primary care clinicians disliked rigid guidelines and did not think that encouraging the use of self-management plans for all patients would be beneficial. Finally, the need for flexible approaches to meet the varying needs of different practices, particularly those that were struggling, was considered.

### Challenge 7: Promoting effective models of leadership

Some of the participants seemed to seek a more traditional model of disease-based leadership by the respected hospital-based specialist who initiated the project. Most, however, recognised the importance of identifying local practice-based champions. This proved difficult:

‘There’s no particular person within primary care who’s standing up and sort of flying a flag for respiratory services.’

(System leader)

In place of this, reference was made to a more dispersed and shared model of leadership that drew on the energy derived from established networks of GPs:

‘You need to identify networks that already exist, because they’ve been there for 20 years … or they went to the same medical school and they’ve been going to the same educational meetings for a long time. So the networks already exist.’

(System leader)

### Challenge 8: Managing change by achieving a balance between positive incentives and sanctions

Without exception, the interviewees regarded financial incentives as an important and powerful lever for changing the behaviours of practitioners. Several people suggested that no improvement initiative would work unless it was aligned to the QOF and that paying people to attend educational courses, or at least covering their costs, was the best way of ensuring attendance. Several of the participants were uncomfortable with this, worried that short-term compliance behaviours were trumping substantive and sustained improvement behaviours, but at the same time they were pragmatic:

‘I don’t particularly like target-led practice because I think you miss a lot out using that, but I think sometimes, at least initially, to get them in the mind-set of having to do something regularly …’

(System leader)

Some people suggested that financial incentives had become necessary in general practice because of an unwillingness or inability on the part of senior managers to manage poor performance in any other way.

### Challenge 9: Embedding change and a commitment to continuous improvement

No references were made in the interviews to the need to embed or sustain improvement arising from the project:

‘I’m a bit worried that when these projects finish … we’ll be left to our own devices again.’

(GP)

### Challenge 10: Considering the side effects of change

Some reference was made to unintended consequences that could arise from purposeful improvement projects. One responder referred to the risk that focusing on one medical condition might damage the holistic care of patients and several people were concerned that an orientation around measurement was not in the patient’s best interest:

‘There’s a lot of bureaucracy but when it comes down to reality it’s the human being that counts and we do see our patients holistically, not as a statistic.’

(GP)

## DISCUSSION

### Summary

This study used a specific COPD improvement project to provide new insights into the nature of large-scale improvement in general practice. A genuine willingness was demonstrated by the practice staff to participate in and learn from well-designed collaborative improvement projects. The 10 challenges for improvement derived largely from hospital-based studies were in the most part applicable to improvement in primary care settings and provided a useful analytical framework. However, at an operational level it is clear that large-scale change in primary care presents some particular challenges, which need to be better understood and addressed if improvement initiatives are to be effective.^13^ We found limitations in the capacities and capabilities of the primary care workforce to undertake systematic improvement, significant problems with access to and the quality of data, and tensions between the narrative-based generalist orientation of many primary care clinicians and the quantitative single-disease orientation that has characterised much of the quality improvement movement to date.

### Strengths and limitations

The study combined empirical data and programme-level theory^14^,^15^ derived from evidence-based guidance to gain insights into the relatively new field of scholarship of improvement at scale in general practice. The study was ambitious in a number of ways: scale; choice of disease and locality; and the complexity of the design and implementation of the intervention. It is possible that additional insights would have been gained by interviewing or surveying a larger sample of project participants, and by using additional sources of data, including group interviews, documentary analysis, and observation. However, the pragmatic choice of a simple evaluative design is justified by the new insights gained.

### Comparison with existing literature

The findings support an emerging body of evidence that improvement requires more than just an effective intervention.^16^ It also requires a deep understanding of and sensitivity to the context within which improvement projects are taking place. This includes the shared values, experiences, and leadership capabilities of the participants, the constraints under which they operate, and the infrastructure supporting them.^17^,^18^ While the importance of context has long been acknowledged by experienced primary care managers and clinicians leading improvement on the ground, the authors found little empirically based analysis in the literature relating specifically to large-scale improvement projects in the general practice sector.

The findings also confirm the pragmatic orientation of many front-line workers,^19^ and builds on this work by demonstrating the need to design and promote new initiatives in a way that both aligns to their professional values and makes their lives easier. The multifaceted nature of the improvement interventions is not only compatible with the prevailing view that single interventions have limited impact,^11^ but also gives more scope for the improvement work to be tailored to the preferences of different professionals.^20^

### Implications for practice

This study presents some potentially useful findings for those leading large-scale quality improvement projects in general practice settings. First, for improvement efforts to operate effectively within the current organisation of primary care services in the UK, greater resource needs to be allocated to communicating, influencing, and engaging practices in the principles and practices of system-based improvement. Interventions, particularly educational ones, need to be more accessible, more timely, and more flexible. Greater attention is required to build the capacity and capability of primary care teams to improve the quality of their data and to utilise these data for improvement purposes.

Second, while much of the evidence derived from the evaluation of improvement initiatives in the acute sector appears to be relevant to general practice, it nevertheless requires adaptation in order to be relevant to primary care settings. In particular, notwithstanding the need for better use of data, it is likely that quality improvement initiatives are more likely to engage many primary care clinicians if they are seen to value both narrative and numeric data.^21^ In addition, it is possible that primary care staff will be more likely to engage if projects move beyond a focus on single conditions, perhaps examining improvement in the growing population of patients with multiple morbidities.^22^

This study raises broader questions about whether new, emerging models of primary care might help to create a more conducive environment for systematic improvement. Many of the problems identified in this evaluation are a consequence of the relative isolation and lack of infrastructure to support improvement in the sector. As practices become larger, and work more closely with neighbouring practices and hospitals through federations and clusters,^23^ improvement initiatives such as the one described in this study are likely to reap greater benefits for patients.
